# MRI and amino acid PET detection of whole-brain tumor burden

**DOI:** 10.3389/fonc.2023.1248249

**Published:** 2023-09-21

**Authors:** Peng Chen, Matthew L. Scarpelli, Debbie R. Healey, Shwetal Mehta, C. Chad Quarles

**Affiliations:** ^1^ School of Health Sciences, Purdue University, West Lafayette, IN, United States; ^2^ Department of Cancer Systems Imaging, The University of Texas (UT) MD Anderson Cancer Center, Houston, TX, United States; ^3^ Ivy Brain Tumor Center, Barrow Neurological Institute, Phoenix, AZ, United States

**Keywords:** MRI, amino acid PET, tissue clearing, glioblastoma, brain tumor, imaging

## Abstract

**Background:**

[^18^F]fluciclovine amino acid PET has shown promise for detecting brain tumor regions undetected on conventional anatomic MRI scans. However, it remains unclear which of these modalities provides a better assessment of the whole brain tumor burden. This study quantifies the performance of [^18^F]fluciclovine PET and MRI for detecting the whole brain tumor burden.

**Methods:**

Thirteen rats were orthotopically implanted with fluorescently transduced human glioblastoma cells. Rats underwent MRI (T1- and T2-weighted) and [^18^F]fluciclovine PET. Next brains were excised, optically cleared, and scanned ex vivo with fluorescence imaging. All images were co-registered using a novel landmark-based registration to enable a spatial comparison. The tumor burden identified on the fluorescent images was considered the ground truth for comparison with the *in vivo* imaging.

**Results:**

Across all cases, the PET sensitivity for detecting tumor burden (median 0.67) was not significantly different than MRI (combined T1+T2-weighted) sensitivity (median 0.61; p=0.85). However, the combined PET+MRI sensitivity (median 0.86) was significantly higher than MRI alone (41% higher; p=0.004) or PET alone (28% higher; p=0.0002). The specificity of combined PET+MRI (median=0.91) was significantly lower compared with MRI alone (6% lower; p=0.004) or PET alone (2% lower; p=0.002).

**Conclusion:**

In these glioblastoma xenografts, [^18^F]fluciclovine PET did not provide a significant increase in tumor burden detection relative to conventional anatomic MRI. However, a combined PET and MRI assessment did significantly improve detection sensitivity relative to either modality alone, suggesting potential value in a combined assessment for some tumors.

## Introduction

Imaging has a critical role in brain tumor localization, treatment planning and response assessment. Currently, conventional magnetic resonance imaging (MRI), including T2-weighted (T2w) MRI and gadolinium enhanced T1-weighted (T1w) MRI, are utilized routinely throughout brain tumor patient care (1). While these MRI methods give a useful assessment of tumor-induced blood-brain barrier breakdown (T1w MRI) and edema (T2w MRI), their sensitivity for detecting invasive tumor regions without gadolinium enhancement is known to be low (2).

Invasive tumors without gadolinium enhancement have been detected with amino acid positron emission tomography (PET). Various amino acid PET tracers, including anti-1-amino-3-[^18^F]fluorocyclobutane-1-carboxylic acid (fluciclovine), [^18^F]fluoroethyl-l-tyrosine (FET), and [^11^C]methionine (MET) have been used in brain tumors. These amino acid PET tracers cross the blood brain barrier (BBB), making them potentially more sensitive to the invasive non-enhancing tumor regions than MRI (3). Indeed, these amino acid PET methods have been shown to detect some non-enhancing brain tumor regions (4–6). However, despite wide-spread use, the overall performance of amino acid PET and conventional MRI for detecting the whole-brain tumor burden has not been quantified. This gap in knowledge, makes it challenging to know when one, or both, imaging modalities should be utilized. Given the relatively large costs associated with these imaging modalities, it would be helpful to have a strategy for when each modality, or both, should be utilized. Further, without fully quantifying detection performance, it is unknown how potential limitations in MRI and PET may negatively impact patient care.

Previous evaluations of MRI and PET performance in brain tumors utilize cross-validation of imaging findings with surgical sampling (6). These studies have provided a comparison of MRI and PET findings with surgical samples at select locations. However, surgical sampling does not provide comparisons across the entire brain, meaning the performance of MRI and PET for detecting whole-brain tumor burden remains unknown. This study takes initial steps in addressing this gap in knowledge.

The study utilizes an innovative multimodal image registration method, facilitating a spatial comparison of MR/PET images with ex vivo optical images across the whole rodent brain (7). This is achieved through optical tissue clearing, which eliminates light penetration limitations that have previously restricted optical imaging to thin histologic sections. It enables a spatial comparison of *in vivo* PET and MRI findings with ex vivo optical images of tumor burden. These results are significant, as all patients with high-grade brain tumors will undergo some form of medical imaging. Quantifying the performance of these imaging methods would improve our ability to interpret imaging findings and make reliable decisions regarding patient care.

## Materials and methods

### Animals and disease models

This study utilized Charles River’s (Wilmington, MA) immunodeficient Rowett Nude (RNU) rats. Four patient-derived human glioblastoma cell lines were utilized in this study and provided by the Biobank Core Facility at Barrow Neurological Institute at St. Joseph’s Hospital and Medical Center. These cell lines were harvested from fresh isolates of four different patients with pathologically verified glioblastoma at Barrow Neurological Institute. All PDX tumors demonstrated invasiveness and genetic heterogeneity similar to human glioblastoma. This resulted in utilization of four different patient-derived xenograft (PDX) glioblastoma models for this study (termed GB7, GB126, GB94, GB187). Two of the models were derived from primary glioblastoma (GB7 and GB94) and two were derived from recurrent glioblastoma (GB187 and GB126). All PDXs were deidentified prior to use in the study to conform with the Biobank Core Facility’s Institutional Review Board protocol. For comparison, two human brain tumor glioblastoma long-term cultured cell lines (U87 and U251) were also utilized in these studies (8, 9).

Prior to implantation, cell lines were transduced with a lentivirus expressing luciferase and tdTomato genes to enable bioluminescent and fluorescent tumor cell imaging, respectively. For implantation, cells were harvested and resuspended in PBS at a concentration of 125 million cells/mL. Four microliters of cell suspension were then orthotopically implanted into five weeks old RNU rats. Tumor cells were implanted 0.3 mm posterior to the bregma and 3.5 mm to the right of the sagittal suture at a depth of 4.5 mm using the StereoDrive stereotaxic system (Neurostar). After implantation, tumor growth was tracked using bioluminescent imaging with an *In Vivo* Imaging System (IVIS, PerkinElmer). The St. Joseph Hospital and Medical Center’s Institutional Animal Care and Use Committee approved of all experimental procedures performed in this study and all animals were treated humanely in accordance with the Laboratory Animal Welfare Act.

### *In vivo* MRI and PET

*In vivo* MRI and PET scanning were performed once IVIS bioluminescence signals averaged greater than 10^7^ photons/sec from tumor regions. PET scanning was performed using a Bruker Albira Si 3 ring preclinical PET scanner. Approximately 12 MBq of fluciclovine was injected intravenously at the tail vein for each rat prior to PET scans. PET scans were acquired from 35 to 55 minutes post-injection of tracer. For all PET scans the brain was positioned at the center of the field of view. An ordered subset expectation maximization algorithm was used for PET image reconstruction. Reconstructed PET images included corrections for scatter, deadtime, and decay of tracer. The fluciclovine amino acid tracer was used for these PET scans as it has demonstrated a higher tumor-to-background image contrast than other amino acid tracers (4, 5) and it is widely available commercially in the United States.

MRI scanning was performed immediately following completion of the PET scan. MRI was performed with a 7-Tesla Bruker BioSpec preclinical MRI scanner. Animals were kept sedated when transferred between PET and MRI scanners and remained positioned on the same Bruker multimodality rat bed. Conventional anatomic T1- and T2-weighted MR images were acquired. The T2w scan included rapid acquisition with relaxation enhancement (RARE), sequence with a repetition time of 6,500 ms, an effective echo time of 50 ms, and a voxel size of 0.2 × 0.2 × 0.5 mm3. The T1w pre and T1w post scan included a Fast Low Angle SHot (FLASH) sequence with a repetition time of 16 ms, echo time of 2 ms, and voxel size of 0.2 × 0.2 × 0.5 mm3. Gadolinium contrast agent was injected intravenously between the T1w pre and T1w post scans.

### Tissue preparation

Following *in vivo* PET and MRI, rats were sacrificed via transcardiac perfusion with 150 mL of 100 U/mL heparinized phosphate buffer (PB) to clear the blood from the system. This procedure was followed by 4% paraformaldehyde (PFA) to fix the tissue (300 mL). Ten minutes prior to the perfusion, 500ul of 1mg/mL Lycopersicon Esculentum (Tomato) Lectin (LEL, TL), DyLight™ 649 (DL-1178-1) was injected via tail vein. Once perfusion was complete, the brain was dissected and immersed in 4% PFA for an additional 24-36 hours to complete the fixation process. After immersion in PFA, the tissue was washed with 0.1-M PB and stored in 0.1-M PB.

### *Ex vivo* MRI and tissue slicing

Next, ex vivo MRI was performed on the excised rodent brains to enable registration of *in vivo* and ex vivo images (7). During the MRI scan whole brains were secured within a pathology slice block (Acrylic Brain Slicer Matrix, Zivic Instruments, Pittsburgh, Pennsylvania) and placed within a cylindrical tube filled with PB. The MRI acquisition was set so that the MRI slices were aligned parallel to the slices of the pathology slice block. Ex vivo MRI included the same parameters as the *in vivo* MRI. Immediately following MRI, the slice block with the brain was removed from the cylindrical tube, and the brain was sliced into 1-mm coronal slices. Each brain slice was then placed in 0.1-M PB in preparation for optical clearing. The brains were sliced into 1-mm slices because it was the thinnest size available in the acrylic MRI-compatible slice block.

### Optical clearing

A clear, unobstructed brain imaging cocktail (CUBIC)-based protocol was utilized in clearing the tissue slices (7). Following clearing tissue slices were washed and immersed in a primary antibody solution consisting of 0.1M PB containing 0.1% TritonX-100 (Sigma), 1% normal goat serum (Sigma), and a 1:250 dilution of ASCT2 (D7C12) Rabbit mAb (8057S, Cell Signaling) for 3 days at 4°C with orbital shaking. After the primary incubation, the tissue was washed and transferred to a secondary antibody solution containing 0.1% TritonX-100 (Sigma), 1% normal goat serum (Sigma), and Goat anti-Rabbit IgG (H+L) Cross-Adsorbed Secondary Antibody, Alexa Fluor™ 488 (A-11008, Invitrogen) at a dilution of 1:400 for 3 days at 4oC with orbital shaking. When the secondary incubation was completed, the tissue was washed once again, followed by immersion in EasyIndex (LifeCanvas Technologies) for 1 hour at 37°C with a rocking motion. All washes were performed three times for 2 hours in 0.1M PB at room temperature on a rocking platform.

### *Ex vivo* fluorescence imaging

Optically cleared brain slices were imaged using an IVIS Spectrum (PerkinElmer, Waltham, Massachusetts). For IVIS imaging, each set of brain slices were placed in a 12-well tissue culture plate filled with EasyIndex for refractive index matching (LifeCanvas Technologies). For anti-ASCT2 FITC imaging, an excitation wavelength of 500 nm and an emission wavelength of 540 nm was used. For tdTomato imaging, an excitation wavelength of 570 nm and an emission wavelength of 620 nm were used. For lectin imaging, an excitation wavelength of 640 nm and an emission wavelength of 680 nm was used. All IVIS imaging consisted of a 60-second acquisition with a bin size of 1 (corresponding to 34.4 μm pixels), F/Stop of 8, and field of view of 6.6 cm × 6.6 cm. Two acquisitions per brain were required to ensure the field of view covered the entire 12-well plate. The resulting fluorescence images have units of radiant efficiency (i.e. photons/s/unit area/unit steradian/wattage of excitation laser).

### Image registration and segmentation

All registrations and segmentations were carried out utilizing 3D Slicer v4.11.20210226 (Cambridge, MA). *In vivo* MRI and PET images were registered to ex vivo MRI images using affine registration. The fluorescence images of optically cleared brain slices were registered slice by slice to the ex vivo MRI slices using a landmark-based registration (7). After registration, all images were in the reference frame of the ex vivo MRI with slices of the same thickness. Then, tumor volumes were segmented individually on T1w MRI, T2w MRI, PET, and tdTomato fluorescence images using a semi-automatic threshold-based technique. For all segmentations a threshold was manually selected for each tumor using Slicer’s built in Threshold tool in the Segment Editor module. Regions there were clearly not tumor, such as the hyperintense ventricles on the T2w MRIs or areas outside the brain, were manually removed. In addition, the T1w and T2w MRI segmentations were combined to give an overall MRI segmentation. An additional expanded MRI segmentation was created by expanding the MRI segmentation by 2 mm.

### Image and statistical analysis

In order to determine the performance of MRI and PET for detecting brain tumors, we spatially compared the MRI and PET tumor segmentations with the tumor segmentations derived from the fluorescently labelled tumor images (i.e., tdTomato images). For MRI, this comparison included calculating the sensitivity, dice similarity coefficient, and maximum surface distance between the MRI and tdTomato tumor segmentations. The sensitivity was calculated for each tumor by dividing the number of voxels included in both the MRI segmentation and tdTomato segmentation by the total number of voxels in the tdTomato segmentation. Specificity was calculated for each tumor by taking the number of voxels included in the optical brain region minus the number of voxels within the union of the MRI tumor segmentation and optical tumor segmentation divided by the number of voxels included in the optical brain region minus the number of voxels in the optical tumor segmentation. The surface distance was calculated by determining the minimum distance between a given surface voxel and any voxel on the surface of the other segmentation. This surface distance calculation was made for each voxel on the surface of the MRI and tdTomato tumor segmentations. It was then summarized for each tumor by taking the maximum value (to assess a ‘worst-case’ scenario). Similar comparisons were made for the PET and tdTomato tumor segmentations. For all the aforementioned calculations, the *in vivo* images (MRI and PET) were upsampled to match the voxel size of the ex vivo tdTomato images. MATLAB was used for this analysis. Non-parametric Wilcoxon tests were used to evaluate differences between groups and P-values less than 0.05 were considered significant.

In addition, to determine the relationship between fluciclovine PET uptake and underlying physiologic quantities, we correlated fluciclovine uptake with optical imaging measurements. On a tumor-level, the maximum tumor PET uptake and mean tumor fluorescence intensities (tdTomato, lectin, and ASCT) were compared. All imaging values were normalized by dividing tumor uptake by the mean contralateral normal brain values. In addition, we compared the PET uptake and fluorescence intensities in the tumor on a voxel-level. For this comparison, the fluorescence images were downsampled to the PET voxel size (0.5x0.5x1 mm). For both the tumor-level and voxel-level analyses, a multivariate analysis was performed by generating linear regression models with PET uptake as the dependent variable and the fluorescence intensity values as the independent variables. Additional independent variables included the gadolinium enhancement status (derived from MRI) and tumor volume. Before being inserted into the multivariate regression, the data were log-transformed and standardized (10). All statistical analysis, including multivariate modelling was performed with the Stata/SE 17.0.

## Results

This study included 13 rats implanted with one of six different human tumor xenografts (U87, U251, GB7, GB94, GB126, or GB187). The full results for each individual tumor xenograft are shown in in the **Table 1**.

**Table 1:** Individual results for each tumor xenograft.

	Xenograft cell line	Tumor Volume (mm^3^)						Sensitivity								Specificity					Dice Coefficient									Maximum surface distance (mm)								
		Optical	T1	T2	T1 and T2	PET	MRI+PET	T1	T2		T1 and T2		PET		MRI+PET	T1	T2	T1 and T2	PET	MRI+PET	T1	T2		T1 and T2		PET		MRI+PET		T1	T2		T1 and T2		PET		MRI+PET	
**Gd+** **N=4**	GB126	161	122	101	128	185	191	0.74	0.62		0.77		0.87		0.91	0.99	1	0.99	0.91	0.91	0.84	0.76		0.86		0.81		0.83		1.6	1.8		1.6		2.0		2.0	
	GB126	86	61	52	63	112	117	0.68	0.59		0.70		0.84		0.89	1	1	1	0.93	0.93	0.80	0.74		0.81		0.73		0.75		1.1	1.1		1.1		2.0		2.0	
	GB126	124	69	58	81	151	153	0.53	0.44		0.61		0.90		0.91	0.99	0.99	0.99	0.92	0.92	0.68	0.60		0.74		0.81		0.81		2.6	2.9		2.6		1.8		1.8	
	U87	104	89	122	133	211	226	0.76	0.79		0.84		0.93		0.95	0.99	0.94	0.93	0.82	0.80	0.82	0.72		0.74		0.61		0.60		1.2	2.1		2.1		2.3		2.3	
**Gd+ Tumors Median**		**114**	**79**	**80**	**105**	**168**	**172**	**0.71**	**0.61**		**0.74**		**0.89**		**0.91**	**0.99**	**0.99**	**0.99**	**0.91**	**0.91**	**0.81**	**0.73**		**0.78**		**0.77**		**0.78**		**1.4**	**2.0**		**1.9**		**2**		**2**	
**Gd-** **N=9**	GB187	104	0	72	72	161	172	0	0.53		0.53		0.85		0.89	1	0.97	0.97	0.88	0.87	0	0.63		0.63		0.67		0.67		nd	2.7		2.7		2.8		2.8	
	GB187	76	0	60	60	87	109	0	0.52		0.52		0.56		0.73	1	0.96	0.96	0.91	0.90	0	0.58		0.58		0.53		0.60		nd	3.2		3.2		3.1		3.1	
	GB187	30	0	28	28	29	47	0	0.73		0.73		0.46		0.88	1	0.97	0.97	0.93	0.91	0	0.76		0.76		0.47		0.69		nd	1.2		1.2		2.0		2.0	
	GB187	63	0	60	60	69	89	0	0.63		0.63		0.67		0.80	1	0.96	0.96	0.95	0.93	0	0.64		0.64		0.63		0.66		nd	1.8		1.8		2.4		2.4	
	U251	64	0	94	94	120	133	0	0.71		0.71		0.82		0.86	1	0.89	0.89	0.84	0.82	0	0.58		0.58		0.57		0.56		nd	2.4		2.4		2.4		2.4	
	U251	22	0	8	8	0	8	0	0.24		0.24		0		0.24	1	0.99	0.99	1	0.99	0	0.35		0.35		0		0.35		nd	3.1		3.1		nd		3.1	
	GB7	132	0	71	71	0	71	0	0.37		0.37		0		0.37	1	0.97	0.97	1	0.97	0	0.47		0.47		0		0.47		nd	7.6		7.6		nd		7.6	
	GB7	131	0	39	39	0	39	0	0.21		0.21		0		0.21	1	0.98	0.98	1	0.98	0	0.32		0.32		0		0.32		nd	8.6		8.6		nd		8.6	
	GB94	20	0	57	57	0	57	0	0.35		0.35		0		0.35	1	0.93	0.93	1	0.93	0	0.18		0.18		0		0.18		nd	4.7		4.7		nd		4.7	
**Gd- Tumors Median**		**64**	**0**	**60**	**60**	**29**	**71**	**0**	**0.52**		**0.52**		**0.46**		**0.73**	**1**	**0.97**	**0.97**	**0.93**	**0.93**	**0**	**0.58**		**0.58**		**0.47**		**0.56**		**nd**	**3.1**		**3.1**		**2.4**		**3.1**	
**Overall Median**		**95**	**0**	**60**	**67**	**87**	**110**	**0**	**0.56**		**0.61**		**0.67**		**0.87**	**1**	**0.97**	**0.97**	**0.93**	**0.92**	**0**	**0.62**		**0.64**		**0.57**		**0.63**		**1.4**	**2.6**		**2.5**		**2.2**		**2.4**	

Optical, tdTomato optical images; T1, gadolinium-enhanced t1-weighted MRI; T2, t2-weighted MRI; MRI, combine t1 and t2-weighted MRI; PET, Amino acid PET; MRI+PET, combine t1 and t2-weighted MRI and PET; nd, entire tumor not detected (not included in median calculations for surface distance); Gd+, Gadolinium enhancing tumor (N=4); Gd-, Gadolinium non-enhancing tumor (N=9, PET-detected: N=5, PET-undetected: N=4).

### Performance of MRI

All tumors with gadolinium enhancement were visually apparent on T1w MRI (n=4) and none of the tumors without gadolinium enhancement were visible on T1w MRI (n=9). All tumors were visible on T2w MRI (n=13). The overall MRI (combined T1w and T2w MRI) measured tumor volume (median 63 mm^3^) was smaller than the tumor volume measured with tdTomato fluorescence imaging (median 86 mm^3^; p=0.13).

In gadolinium enhancing tumors, the median sensitivity of T1w MRI for detecting tdTomator positive tumor voxels was 0.71 (range 0.53 – 0.76). Similarly, the median T2w MRI sensitivity for detecting tumor voxels in gadolinium enhancing tumors was 0.61 (range 0.44 – 0.79). In tumors without gadolinium enhancement, the median T2w MRI sensitivity was 0.52 (range 0.21 – 0.73). The overall (combined T1+T2w) MRI sensitivity for detecting tdTomato positive tumor voxels was not significantly different between tumors without gadolinium enhancement (median sensitivity 0.52) and tumors with gadolinium enhancement (median sensitivity 0.74; p=0.05).

In tumors with gadolinium enhancement, the median specificity of T1w MRI was 0.99 (range 0.99 - 1) and for T2 MRI was also 0.99 (range 0.94 - 1). For gadolinium non-enhancing tumors, the median specificity of T2w MRI was 0.97 (range 0.89 - 0.99). The overall (combined T1+T2w) MRI specificity was not significantly different between gadolinium non-enhancing tumors (median value 0.97) and gadolinium enhancing tumors (median value 0.99; p=0.18).

**Figure 1:**
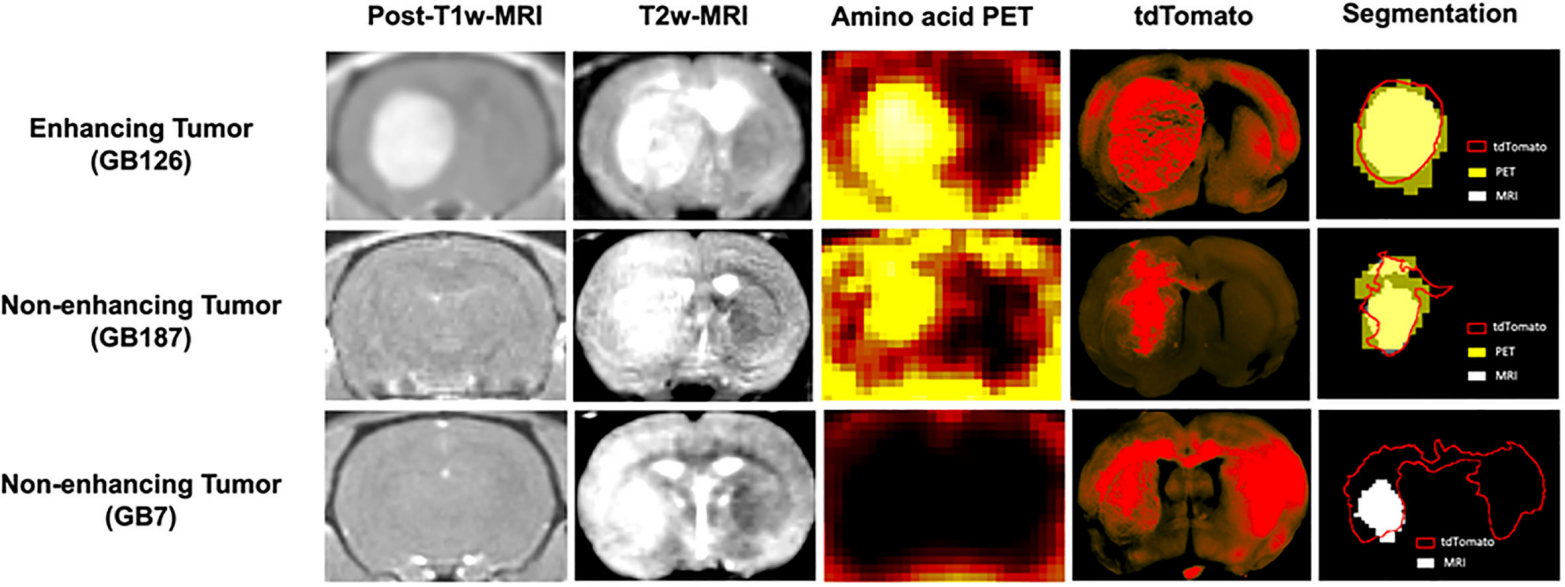
Coronal brain images showing post contrast-T1-weighted-MRI, T2-weighted-MRI, amino acid PET, tdTomato optical imaging, and tumor segmentations. The top row shows a gadolinium enhancing tumor (GB126). The middle row shows a gadolinium non-enhancing tumor detected with PET (GB187). The bottom row shows a gadolinium non-enhancing tumor not detected with PET (GB7).

The two GB7 PDXs were some of the most difficult to detect and went undetected on T1w MRI. Although these tumor xenografts were visible on T2w MRI, tumor invasion into the contralateral brain hemisphere went undetected on all imaging modalities (**Figure 1**). This resulted in low sensitivity and high maximum surface distance between the tdTomato and MRI tumor segmentations (median surface distance = 8.1 mm).

### Performance of PET and combined PET+MRI

All four gadolinium enhancing tumors were visible on PET. Five out of the nine tumors without gadolinium enhancement were visible on PET. **Tables 2** **–** **4** summarize quantitative results, for gadolinium enhancing tumors (**Table 2**), non-enhancing tumors visible on PET (**Table 3**) and non-enhancing tumors not visible on PET (**Table 4**). For tumors visible on PET (n=9), the PET-detected tumor volume (median 120 mm^3^) was larger than the tumor volume detected with tdTomato optical imaging (median 86 mm^3^; p=0.008) and MRI (median 72 mm^3^, p=0.004).

**Table 2:** Median values and ranges for gadolinium enhancing tumors (n=4).

	Tumor Volume (mm^3^)		Sensitivity		Specificity		Dice coefficient		Max surface distance (mm)	
**T1 MRI**	79	(61, 122)	0.71	(0.53, 0.76)	0.99	(0.99, 1)	0.81	(0.68, 0.84)	1.4	(1.1, 2.6)
**T2 MRI**	79	(52, 122)	0.61	(0.44, 0.79)	0.99	(0.94, 1)	0.73	(0.60, 0.76)	2.0	(1.1, 2.9)
**T1 and T2 MRI**	104	(63, 133)	0.74	(0.61, 0.84)	0.99	(0.93, 1)	0.77	(0.74, 0.86)	1.8	(1.1, 2.6)
**PET**	168	(112, 211)	0.89	(0.84, 0.93)	0.91	(0.82, 0.93)	0.77	(0.61, 0.81)	2.0	(1.8, 2.3)
**MRI+ PET**	172	(117, 226)	0.91	(0.89, 0.95)	0.91	(0.80, 0.93)	0.78	(0.60, 0.83)	2.0	(1.8, 2.3)
**Optical (tdTomato)**	114	(86, 161)								

**Table 3:** Median values and ranges for gadolinium non-enhancing tumors visible on PET (n=5).

	Tumor Volume (mm^3^)		Sensitivity		Specificity		Dice coefficient		Max surface distance (mm)	
**T2 MRI**	60	(28, 94)	0.63	(0.52, 0.73)	0.96	(0.89, 0.97)	0.63	(0.58, 0.76)	2.4	(1.2, 3.2)
**PET**	87	(29, 161)	0.67	(0.46, 0.85)	0.91	(0.84, 0.95)	0.57	(0.47, 0.67)	2.4	(2.0, 3.1)
**T2 MRI and PET**	109	(47, 172)	0.86	(0.73, 0.89)	0.90	(0.82, 0.93)	0.66	(0.56, 0.69)	2.4	(2.0, 3.1)
**Optical (tdTomato)**	64	(30, 104)								

**Table 4:** Median values and ranges for gadolinium non-enhancing tumors not visible on PET (n=4).

	Tumor Volume (mm^3^)		Sensitivity		Specificity		Dice coefficient		Max surface distance (mm)	
**T2 MRI**	48	(8, 71)	0.30	(0.21, 0.37)	0.97	(0.93, 0.99)	0.34	(0.18, 0.47)	6.2	(3.1, 8.6)
**Optical (tdTomato)**	77	(20, 132)								

Overall, across all 13 tumors, the PET sensitivity (median 0.67) was not significantly different than the MRI (combined T1 and T2) sensitivity (median 0.61; p=0.85). Similarly, the PET specificity (median 0.93) was not significantly different than MRI (median 0.97, p=0.08).

Overall, across all 13 tumors, the combined MRI+PET sensitivity (median 0.86) was significantly higher than MRI alone (p=0.004) or PET alone (p=0.0002). Whereas the specificity of combined PET+MRI (median=0.91) was significantly lower compared with MRI alone (p=0.004) or PET alone (p=0.002). The imaging modalities performed the worst for the tumors in **Table 4**, with the sensitivity of combined PET+MRI less than 0.40 in all four tumors.

**Supplementary Table 1** shows the performance after expanding the MRI (combined T1w and T2w) segmentations by 2 mm. In general, this expansion of the MRI segmentation led to increased sensitivity but reduced specificity. However, in some cases, even the expanded MRI segmentations did not have higher sensitivity than the combined PET+MRI segmentations. In every case, the expanded MRI segmentations had lower specificity than the combined PET+MRI segmentations. This suggests the addition of PET is not the equivalent of simply expanding the MRI segmentations.

### PET correlates

**Figure 2:**
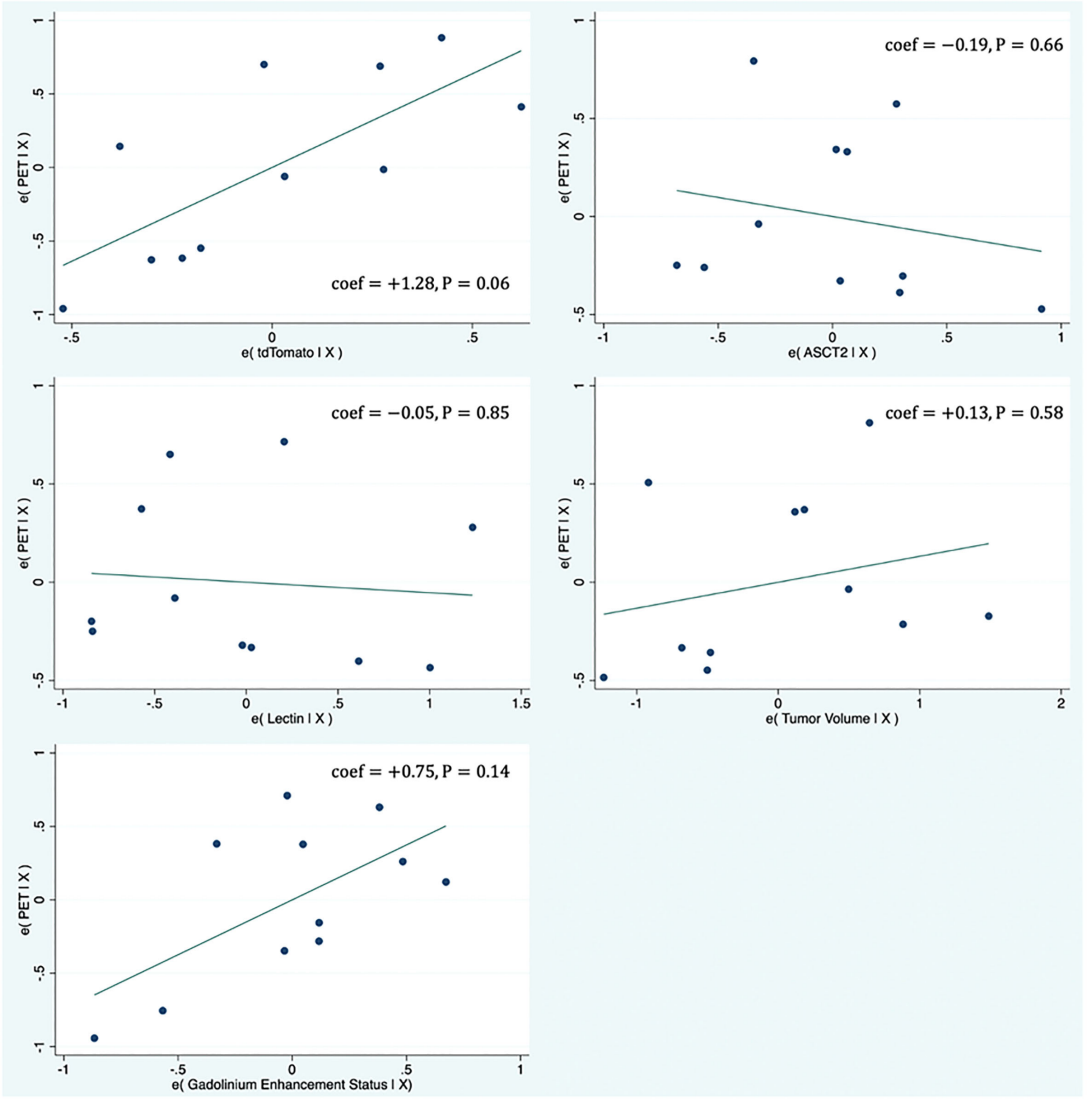
Partial regression plots showing the linear relationship between the PET uptake and various biologic measurements (tdTomato, ASCT2, Lectin, tumor volume, gadolinium enhancement status). Each data point represents measurements summarized for a single tumor. The linear regression line has intercept of zero and a slope equal to the multivariate linear regression coefficient tabulated in **Supplementary Table 1**. The vertical axis in these plots represents the calculated residuals from a linear regression model with the PET uptake as a function of all other independent variables except the independent variable of interest. The horizontal axis represents the calculated residuals from a linear regression model with the independent variable of interest as a function of all the other independent variables.

**Supplementary Table 2** shows the results of the multivariate regression where tumor PET uptake is regressed against various biologic measurements (tdTomtato, ASCT2 amino acid transporter, lectin, tumor volume, and gadolinium enhancement status). The model provided a good fit with R^2^ = 0.77 and the highest model coefficient being tdTomato (β_TDT_ = +1.27; P=0.06). **Figure 2** shows partial regression plots indicating the relationship between the tumor PET uptake and the measured biologic variables in the regression model. When lectin measurements were not included in the model (two tumors did not have lectin measurements), similar results were obtained. Similar results were also found for the multivariate model on a voxel-level, including tdTomato having the strongest association with the PET uptake (β_TDT_ = +0.68; P<0.001). Plots showing the univariate relationship between PET uptake and various biologic measurements (tdTomato, ASCT2, lectin, tumor volume, gadolinium enhancement status) are shown in **Supplementary Figure 1**.

## Discussion

Conventional anatomic MRI scans are widely used in brain tumor patient care. However, the poor sensitivity of conventional MRI for detecting invasive non-enhancing tumor regions may lead to inadequate treatment planning and response assessment. Combining MRI with amino acid PET could help to overcome the limitations of conventional MRI. This study quantified the performance of MRI, amino acid PET, and combined MRI and PET for assessing brain tumors.

Interestingly, when MRI and PET assessments were combined, the sensitivity for detecting tumor burden increased relative to either MRI alone or PET alone, indicating potential benefit in a combined assessment. Most of the gains in sensitivity when adding the PET to MRI were due to detection of tumor regions at the boundary of the tumor, rather than detection of distant tumor foci. However, this combined PET and MRI assessment also led to decreased specificity. This suggests there is a tradeoff between sensitivity and specificity that must be considered when deciding to utilize either combined PET and MRI or a single modality for brain tumor assessment. Generally, the data in **Table 1** suggest the increases in sensitivity for the combined PET and MRI assessment, relative to either modality alone, were greater in tumors without gadolinium enhancement. In addition, the specificity of the combined PET and MRI assessment, decreased less (relative to single modality) in tumors without gadolinium enhancement. This suggests a combined PET/MRI assessment may be most beneficial for tumors or tumor regions without gadolinium enhancement. However, the clinical context also may help to determine whether superior specificity or sensitivity is preferred. For example, in the context of surgical or radiotherapy planning, higher specificity may be preferred so as to not treat normal brain regions. On the other hand, surveillance scans following treatment may prioritize sensitivity so as to reliably quantify treatment effects.

It was observed that MRI-detected brain tumor burden was, in general, smaller than ‘true’ tumor burden (detected with optical imaging). Whereas PET-detected tumor burden was, in general, larger than the ‘true’ tumor burden. A prior study of four PET/MRI scans of a patient with cerebral metastases found fluciclovine PET tumor volumes were larger than contrast enhanced T1-MRI tumor (11), which is consistent with our findings. The increased tumor volume measured by PET is likely due to a combination of two factors, including improved PET sensitivity relative to MRI and PET partial volume effects. The relatively large partial volume effect inherent to PET images decreases spatial resolution and causes a blurring of tumor boundaries, which can lead to diminished specificity. Another issue which may confound these assessments is the possibility of some tumor cells losing their fluorescence. Although we have not observed evidence supporting this in our preclinical studies, it cannot be ruled out.

A prior study compared gadolinium contrast-enhanced T1w MRI and fluciclovine PET tumor detection with surgical specimens. This prior study assessed a total of 37 biopsy locations from five grade IV glioma patients. They reported MRI had a sensitivity of 81.3% for detecting glioma regions, whereas fluciclovine PET/CT had a sensitivity of 90.6% (4). Our findings are similar to this previous study in that we also found a higher sensitivity for PET than T1w MRI in detecting brain tumor regions. These findings highlight the potential complementary value of these two imaging modalities.

An unexpected finding was the relatively high specificity for T2-MRI (overall median value of 0.97). This is unexpected, as clinically T2-MRI is known to detect areas of tumor-induced edema, which lowers its specificity and confounds assessment of tumor burden. Across our preclinical tumor models, few tumors demonstrated significant tumor-induced edema as is characteristically seen on clinical T2-MRI scans. This suggests, these findings have limited applicability in the post radio- or chemo-therapy setting where non-neoplastic T2 changes are commonly observed. In would be beneficial for future work to assess the performance of PET and MRI post-treatment to determine if the findings here might also apply in this context. This lack of edema in PDX brain tumor models has also been observed by other groups (12). It suggests there are limitations to existing brain tumor models in terms of recapitulating the tumor-induced edema observed in clinical cases.

For four of the thirteen tumors in this study, fluciclovine PET did not detect any of the tumor volume. This was unexpected, as it was thought the upregulated amino acid metabolism of brain tumors would lead to fluciclovine tracer uptake. It is possible the tumors relied upon other amino acid transporters besides those which transport fluciclovine, to gain nutrients. It is interesting to note, in both the current study and a prior study (13), a positive correlation was observed between tumor fluciclovine PET uptake and gadolinium enhancement status. The current study also observed fluciclovine uptake is strongly associated with the tumor cell marker tdTomato. Taken together, these results suggest fluciclovine PET uptake is dependent on both a tumor’s blood-brain barrier (i.e., gadolinium enhancement) status and tumor cell content (tdTomato). This may help to explain why four of the tumors in this study were not visible on PET, as these tumors had no evidence of blood-brain barrier breakdown and were diffusely infiltrative with low concentrations of tumor cells. Both of these factors potentially reduce the uptake of the fluciclovine tracer and the detection sensitivity of PET.

In summary, for these glioblastoma xenografts, combining MRI and PET measurements significantly improved detection sensitivity compared to MRI or PET alone. These finding support combined use of PET/MRI scanning in brain tumors. However, the poor performance of both MRI and PET in some glioblastoma models studied here, underlies the need for continued development of improved imaging methods for brain tumors.

## Data availability statement

The raw data supporting the conclusions of this article will be made available by the authors, without undue reservation.

## Ethics statement

The studies involving humans were approved by Barrow Neurological Institute Biobank Core Facility’s Institutional Review Board. The studies were conducted in accordance with the local legislation and institutional requirements. The human samples used in this study were acquired from primarily isolated as part of your previous study for which ethical approval was obtained. Written informed consent for participation was not required from the participants or the participants’ legal guardians/next of kin in accordance with the national legislation and institutional requirements. The animal study was approved by St. Joseph Hospital and Medical Center’s Institutional Animal Care and Use Committee. The study was conducted in accordance with the local legislation and institutional requirements.

## Author contributions

PC contributed to the analysis and interpretation of data. MS contributed to the experimental design, its implementation, analysis, and interpretation of the data. DH contributed to the experimental design, implementation, and interpretation. SM contributed to the experimental design and interpretation of the data. CQ contributed to the experimental design and interpretation of the data. All authors contributed to the article and approved the submitted version.
