# Supplementary material for: MRI and amino acid PET detection of whole-brain tumor burden
Source: Front Oncol. 2023 Sep 21;13:1248249. doi: 10.3389/fonc.2023.1248249 (PMC10558180; doi:10.3389/fonc.2023.1248249)
Supplement: Supplementary file 1 [file DataSheet_1.docx]

**Supplementary Table 1:** *Results for the expansion of the MRI (combined T1 and T2) tumor segmentations by 2 mm.*

|  | **Xenograft cell line** | **Tumor Volume (mm^3^)** | **Sensitivity** | **Specificity** | **Dice Coefficient** | **Maximum surface distance (mm)** |
| --- | --- | --- | --- | --- | --- | --- |
| **Gd+**  **N=4** | GB126 | 374 | 1.00 | 0.57 | 0.60 | 2.3 |
|  | GB126 | 214 | 0.86 | 0.75 | 0.50 | 2.1 |
|  | GB126 | 200 | 0.72 | 0.77 | 0.55 | 2.6 |
|  | U87 | 444 | 0.89 | 0.46 | 0.34 | 4.1 |
| **Gd+ Tumors Median** | | 294 | 0.87 | 0.66 | 0.52 | 2.4 |
| **Gd-**  **N=9** | GB187 | 315 | 0.87 | 0.62 | 0.43 | 2.8 |
|  | GB187 | 330 | 0.95 | 0.50 | 0.36 | 3.0 |
|  | GB187 | 139 | 1.00 | 0.52 | 0.36 | 2.6 |
|  | GB187 | 286 | 1.00 | 0.61 | 0.36 | 3.4 |
|  | U251 | 336 | 1.00 | 0.36 | 0.32 | 4.2 |
|  | U251 | 83 | 0.86 | 0.87 | 0.36 | 2.9 |
|  | GB7 | 363 | 0.70 | 0.59 | 0.37 | 5.7 |
|  | GB7 | 247 | 0.56 | 0.69 | 0.40 | 6.6 |
|  | GB94 | 358 | 1.00 | 0.56 | 0.10 | 6.3 |
| **Gd- Tumors Median** | | 315 | 0.95 | 0.59 | 0.36 | 3.4 |
| **Overall Median** | | 315 | 0.95 | 0.57 | 0.36 | 2.8 |

**Supplementary Table 2:** *Results from the multivariate linear regressions for predicting PET uptake*

| Multivariate Linear regression | Model Coefficients | P value | R^2^ |
| --- | --- | --- | --- |
| Tumor level  (n=11) | $\boldsymbol{\beta}_{\mathbf{TDT}}\boldsymbol{=+}\boldsymbol{1}\boldsymbol{.}\boldsymbol{28}$  $\beta_{ASCT2}=-0.19$  $\beta_{\mathrm{LECTIN}}=-0.05$  $\beta_{\mathrm{VOL}}=+0.13$  $\beta_{\mathrm{ENH}}=+0.75$ | P=0.06  P=0.66  P=0.85  P=0.58  P=0.14 | 0.77 |
| Tumor level  (n=13; no lectin) | $\boldsymbol{\beta}_{\mathbf{TDT}}\boldsymbol{=+}\boldsymbol{0}\boldsymbol{.}\boldsymbol{71}$  $\beta_{ASCT2}=-0.02$  $\beta_{\mathrm{VOL}}=+0.16$  $\beta_{\mathrm{ENH}}=+0.31$ | P=0.007  P=0.94  P=0.49  P=0.25 | 0.72 |
| Voxel level  (n=7) | $\boldsymbol{\beta}_{\mathbf{TDT}}\boldsymbol{=+}\boldsymbol{0}\boldsymbol{.}\boldsymbol{68}$  $\beta_{ASCT2}=+0.14$  $\beta_{\mathrm{LECTIN}}=-0.34$  $\beta_{\mathrm{VOL}}=+0.10$  $\beta_{\mathrm{ENH}}=+0.22$ | P=0.00  P=0.00  P=0.00  P=0.00  P=0.00 | 0.46 |
| Voxel level  (n=9; no lectin) | $\boldsymbol{\beta}_{\mathbf{TDT}}\boldsymbol{=+}\boldsymbol{0}\boldsymbol{.}\boldsymbol{67}$  $\beta_{ASCT2}=-0.01$  $\beta_{\mathrm{VOL}}=+0.13$  $\beta_{\mathrm{ENH}}=+0.21$ | P=0.00  P=0.57  P=0.00  P=0.00 | 0.53 |


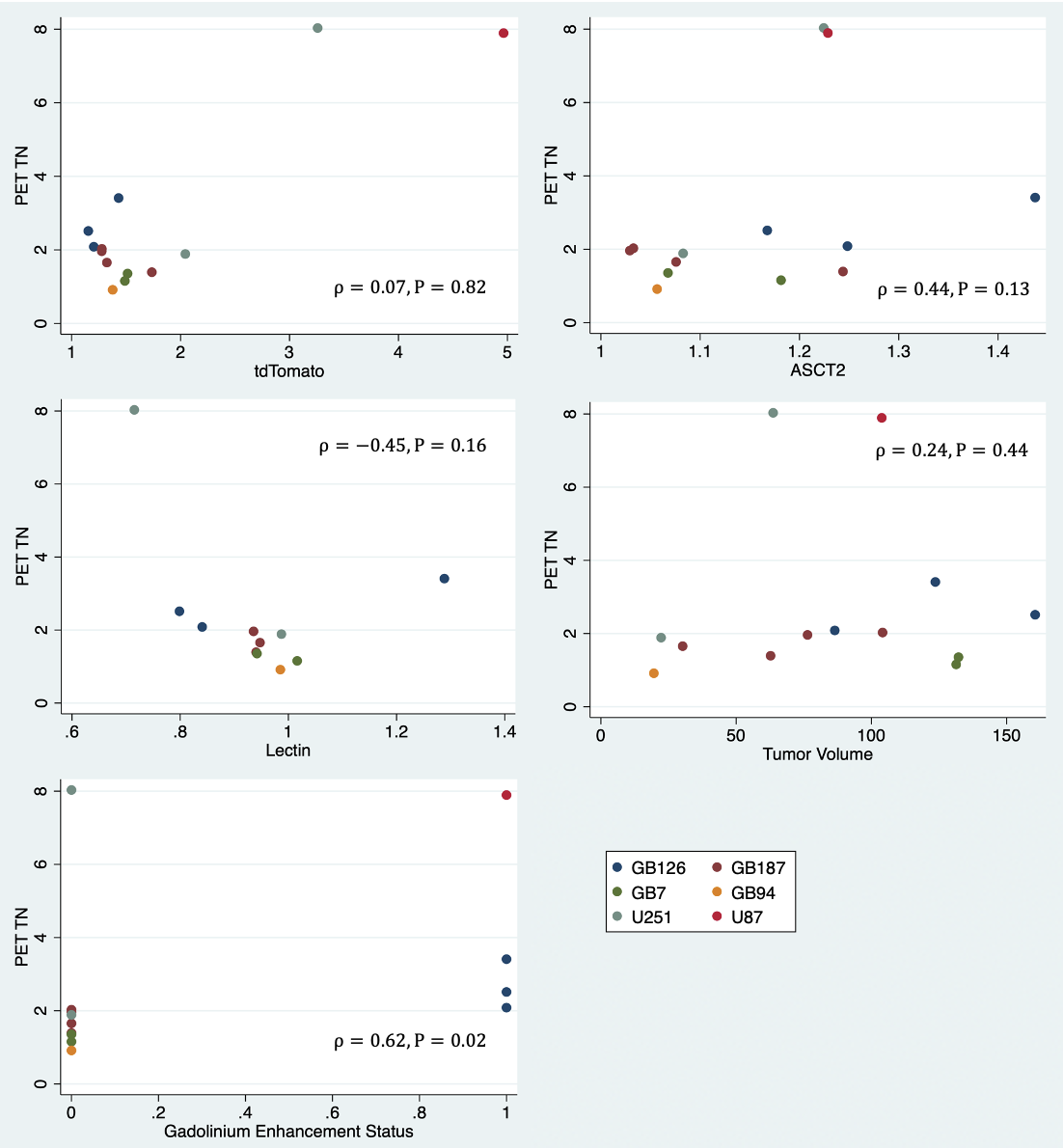


**Supplementary Figure 1:** *Scatter plots showing the univariate relationship between the PET and various biologic measurements (tdTomato, ASCT2, Lectin, tumor volume, gadolinium enhancement status). The Spearman coefficient and P-value are shown for reference. TN = Tumor:Normal brain uptake ratio*
